# Extremely high serum CA19-9 level along with elevated D-dimer in assisting detection of ruptured ovarian endometriosis

**DOI:** 10.1080/07853890.2022.2074534

**Published:** 2022-06-22

**Authors:** Ting Shuang, Yiran Wang, Lanbo Zhao, Kailu Zhang, Panyue Yin, Lin Guo, Wei Jing, Xue Feng, Qiling Li

**Affiliations:** Department of Obstetrics and Gynecology, the First Affiliated Hospital of Xi’an Jiaotong University, Xi'an, China

**Keywords:** CA19-9, D-dimer, CA-125, biomarker, endometriosis

## Abstract

**Background:**

To clarify clinical importance of serum CA19-9, CA-125, and plasma D-dimer (D-D) levels in detecting spontaneously ruptured ovarian endometriosis (OE).

**Materials and Methods:**

We retrospectively examined 173 patients with endometriosis out of 735 cases of OE between 2013 and 2019. Among these, 21 cases were diagnosed as “spontaneously ruptured” after surgery, while the remaining cases were unruptured. Venous blood was collected pre-operatively to detect CA19-9, CA-125, and D-D levels. A receiver operating characteristic curve analysis was applied to test clinical value of each marker.

**Results:**

Among the 21 patients with ruptured OE, 16 had a history of pelvic cysts, 19 claimed sudden onsets of lower abdominal pain, and fluid accumulation were detected in cul-de-sac in only six participants by ultrasound. For serological investigation, both CA19-9 and D-D were significantly elevated in the ruptured OE group (343.09 ± 367.67 U/ml vs. 36.84 ± 40.01 U/ml, 3.39 ± 4.90 mg/L vs. 0.43 ± 0.29 mg/L, both *p* < .0001). The area under curve (AUC) value for the combination of CA19-9 and D-D was 0.975 (95% CI, 0.939 − 0.993), with specificity of 96.69%, and sensitivity of 85.71%. The combination of CA-125, CA19-9 and D-D showed the highest AUC value that up to 0.976 (95% CI, 0.940–0.993), with sensitivity of 95.24%, and specificity of 87.50%.

**Conclusion:**

The combination of CA19-9 and D-D can be chosen as an effective and economical indicators to identify patients with spontaneously ruptured OE in pre-operation assessment. However, from the perspective of differential diagnosis, the combination of CA-125, CA19-9 and D-D is the best choice.

**Key messages**
- Taking into account the economic effect, the combination of CA19-9 and D-D can be chosen as an effective indicators to identify patients with spontaneously ruptured OE in pre-operation assessment.
- From the perspective of differential diagnosis, the combination of CA-125, CA19-9 and D-D is the best choice to identify patients with spontaneously ruptured OE.

## 1. Introduction

Endometriosis, being widely considered as oestrogen-dependence, is defined as the presence of active endometrial glands and stroma tissue outside the uterus. Endometriosis usually causes chronic inflammation and infertility [1–3]. In women of reproductive age, the incidence rate of endometriosis is 5–10%, and for infertile women, the prevalence of endometriosis reaches as high as 50% [4,5]. Ovarian endometriosis (OE) is a common type of endometriosis, occupying 17–44% of total cases [6].

Serum CA19-9 and CA-125 levels are typically applied as biomarkers for malignancies. CA19-9 was initially reported as an antigen associated with colorectal carcinoma and used as a biomarker for potential malignant tumours and metastasis [7]. Lately, it has been reported that the combination of CA19-9, CEA, CA72-4, CA-125, and Ferritin showed promising results in diagnosing colorectal cancer, appraising tumour status, and even evaluating therapeutic effect [8]. Besides, in advanced ovarian cancer, the level of CA19-9 was also elevated [9]. CA-125, another high molecular weight glycoprotein, is used as the first-line screening method for ovarian cancer [10,11]. Recent reports showed that the Symptom Index, along with CA-125 and HE4, had inspiring performance statistics in predicting cancer in women with pelvic masses, with high sensitivity and acceptable specificity [12].

For diagnosis of OE, not only is ultrasonography a very effective and straightforward tool [13], CA-125 and CA19-9 levels have also been recommended as useful markers [14–16]. Ruptured OE is rare. The accuracy for transabdominal ultrasound to diagnose ruptured ovarian cysts was 57.42–88.88%, and the accuracy for intracavitary ultrasound was 68.75–91.89%, the accuracy improved to 91.24–98.79% combining these two methods [17]. However, misdiagnosis still exists. A non-invasive and effective method is needed. As an infrequent gynaecological emergency, ruptured OE is often associated with sudden onset of lower abdominal pain combined with nausea and vomiting, abdominal or uterine tenderness [18]. However, these symptoms are not specific, and it is formidable to discriminate the condition from other gynaecological or non-gynaecologic emergencies. A report claimed the diagnostic rate was as low as 11% for ruptured OE when only symptoms and physical examinations were considered [19]. In patients with endometriosis, Douglas’ pouch is usually tightly closed or adhered. Therefore, in most cases of ruptured OE, typical intraperitoneal fluid and Douglas’ pouch effusion are not able to be observed under ultrasonography [20]. Accordingly, the function of ultrasonography in pre-operative diagnosis of ruptured OE is limited.

Some cases reported extremely elevated serum CA19-9 or CA-125 levels in patients with ruptured OE [18,19,21,22]. Few studies focussed on the relationship between diagnostic efficiency of ruptured OE and abnormally elevated CA-125 or CA19-9 levels [23]. Besides, it is essential to discriminate benign ruptured OE from malignant ovarian tumours [24]. Here in, this report, we aimed to make clear serodiagnosis of abnormally elevated CA-125, CA19-9 along with D-D in assisting pre-operative diagnosis within the context of clinical assessment.

## 2. Materials and methods

### 2.1. Patients

The Ethics Committee of the First Affiliated Hospital of Xi’an Jiaotong University approved our study. All the patients recruited either underwent laparotomy or laparoscopy. The revised classification, issued in 1985 by the American Society for Reproductive Medicine, was applied to diagnose and classify the patients. A total of 735 cases of OE were included between 2013 and 2019. Among them, 21 cases were clarified as “spontaneously ruptured” before the surgery and were collected into the experimental group. The remaining 714 cases were unruptured diagnosed during the surgery. After filtered by inclusion criteria and selected in randomisation, 152 unruptured OE participants were chosen as the control group.

The inclusion criteria were: (i) aged 21–45 years, (ii) underwent surgery *via* either laparotomy or laparoscopic procedure. (iii) diagnosed as OE by postoperative pathology. Exclusion criteria were: (i) combined with malignant ovarian or other tumours, (ii) diagnosed as myoma, adenomyosis, or pelvic inflammatory disease, (iii) had a history of deep venous thrombosis of the lower limbs.

### 2.2. Tumour marker test and coagulation functional test

Venous blood samples were collected from all patients before surgery and tested for serum CA-125 and CA19-9. Coagulation function, including plasma D-D, FIB, was also tested before surgery. Serum CA19-9 and CA-125 levels were measured using Roche electro-chemiluminescence immunoassay (Roche Co., COBAS e 602, Mannheim, Germany) with CA19-9 test Kit (Roche Co., YZB/GER 5395-2014) and CA-125 test Kit (Roche Co., YZB/GER 1568-2015), following manufacturer’s instructions. The normal reference value of CA19-9 was 0–39 U/ml, and that of CA-125 was 0–35 U/ml. Detection of D-D was performed by Latex immunoturbidimetry (SYSMEXM, CS-5100, Tokyo, Japan) using a D-D test kit(SEKISUI Co., GS1-128). The normal reference value of D-D was 0–1.0 mg/L. Those above the upper limit value were considered positive.

### 2.3. Statistical methods

Data were examined using the Statistical Package for the Social Sciences software package (SPSS Statistics ver. 17.0). Levels of CA19-9, CA-125, and D-D, ages of the patients, and diameter of OE were presented as continuous data and were expressed as means with standard deviations (SDs). An unpaired t-test was used to compare individual markers between the experimental and control groups. To clarify the importance of levels of CA-125, CA19-9, and D-D in identifying ruptured OE, a receiver operating characteristic (ROC) curve analysis was applied. The sensitivity, specificity, and positive and negative predictive values were calculated using SPSS Statistics version 17.0. Further, the cut-off value depended on Youden index of each marker was assessed using area under curve (AUC) by MedCal software, version19.05. *P* values less than .05 were considered as statistically significant.

## 3. Results

### 3.1. Measurement of clinical data in the experimental and control groups

A total of 173 patients with OE were identified and retrospectively examined. Among them, 21 patients had spontaneously ruptured OE and were enrolled in the experimental group. Seven patients in the experimental group were classified as early stages (I - II), and the other 15 exhibited advanced disease (III - IV). The remaining 152 unruptured OE cases were defined as the control group. In this group, 59 patients were in early stages, the remaining 93 cases were advanced.

In the experimental group, mean age was 33.0 ± 7.3, that in the control group was 32.2 ± 6.9. The levels of CA-125, CA19-9, D-D, FIB, CA-153, and CA72-4 levels in the experimental and control groups were shown in Table 1.

**Table 1.** Analysis of clinical value in the ruptured OE group and control group.

	Ruptured OE	Unruptured OE	*p* Value
Numbers of patients	21	152	
Age(y) (mean ± SD)	33.0 ± 7.3	32.2 ± 6.9	**NS**
CA-125 (U/ml) (mean ± SD)	852.50 ± 1002.70	70.53 ± 64.49	**.0001**
D-dimer (mg/L) (mean ± SD)	3.40 ± 4.90	0.43 ± 0.29	**.0001**
FIB (g/L) (mean ± SD)	6.41 ± 6.44	2.55 ± 0.58	**.0001**
CA19-9 (U/ml) (mean ± SD)	343.09 ± 367.67	36.84 ± 40.01	**.0001**
Diameter (cm) (mean ± SD)	7.51 ± 3.33	7.07 ± 2.09	**.160**

In the experimental group, the level of CA-125 was significantly elevated compared to that of the control group (852.51 ± 1002.71 U/ml vs. 70.53 ± 64.49 U/ml, *p* < .0001). There was a statistically significant difference in the level of CA19-9 between the experimental group (343.09 ± 367.67 U/ml) and the control group (36.84 ± 40.01 U/ml, *p* < .0001). D-D level and FIB were remarkably increased compared with that of the control group, both with statistically significant difference (3.39 ± 4.90 mg/L vs. 0.43 ± 0.29 mg/L, 6.42 ± 6.44 g/L vs. 2.55 ± 0.58 g/L, respectively, both *p* < .0001).

### 3.2. General information, pre-operative symptoms, and operation findings of ruptured OE

For patients with ruptured OE, we summarized their last medical history, pre-operative data, and performance during the operation. Among the 21 ruptured OE patients, 16 of them ever did ultrasonography and reported pelvic or ovarian cysts. The period from pre-existing ovarian cysts to the onset of lower abdominal pain was arranged from 17 days to 4 years, and the size of tumour was arranged from 4 to 10 cm. Among them, two patients (case 16, case 20) had a history of endometrioma surgery. Nineteen out of the 21 patients had acute lower abdominal pain, accompanied by nausea and vomiting, and appeared abdominal tenderness by physical examination. Four (case 8, 16, 17, 21) out of 19 patients who accepted pelvic examination showed chocolate fluid by culdocentesis (two patients had no sexual experience). Fluid accumulation was detected in the cul-de-sac in six cases (case 6, 8, 10, 16, 17, 21) using ultrasonography. Fever was noted in only one patient with temperature being 39.2 °C. Twenty patients accepted surgery, and the interval from pain attack to surgery arranged from 8 h to 7 days, among which, surgery was performed within 48 h in 11 patients. Only one patient (case 1) accepted selective operation five months after abdominal pain. During the operation, 19 patients were found ruptured ovarian cyst along with chocolate fluid flowing out. Two patients showed haemosiderin staining of the peritoneum, omentum, surface of ovarian cyst, or the intestinal canal (data were shown in Supplemental Table 1). Sixteen patients had laparoscopic surgery and five patients underwent laparotomy. Three patients underwent oophorectomy, and the rest had endometriosis enucleation.

### 3.3. CA-125, CA19-9 levels, and plasma D-D in the experimental group

We subdivided the experimental group into unilateral and bilateral cases. The experimental group was also subdivided into early and advanced disease groups. We then analysed the levels of CA-125, CA19-9, and D-D according to various subgroups (Table 2).

**Table 2.** Level of CA-125, CA19-9 and D-dimer in the ruptured OE group.

	CA-125 (U/ml)	CA19-9 (U/ml)	D-dimer (mg/L)
	(mean ± SD)	(mean ± SD)	(mean ± SD)
Side of occurrence			
Unilateral	884.92 ± 1039.83	369.18 ± 386.25	3.56 ± 5.24
Bilateral	658.00 ± 892.96	186.52 ± 202.10	2.40 ± 2.19
***p* Value**	**0.796**	**0.503**	**0.510**
Stage			
Minimal to mild(I-II)	960.08 ± 907.93	427.65 ± 519.98	1.98 ± 2.18
Moderate to severe (III-IV)	786.31 ± 1087.37	291.05 ± 244.72	4.27 ± 5.92
***p* Value**	**0.962**	**0.271**	**0.242**

Our results showed no statistically significant difference in the levels of CA-125 (884.92 ± 1039.83 U/ml vs. 658.00 ± 892.96 U/ml, *p* = .796), CA19-9 (369.18 ± 386.250 U/ml vs. 186.52 ± 202.10 U/ml, *p* = .503), or D-D (3.56 ± 5.238 mg/L vs. 2.40 ± 2.19 mg/L, *p* = .51) between the unilateral and bilateral subgroups.

When we compared patients with early versus advanced stages, we detected no statistically significant difference in the levels of CA-125, CA19-9, or D-D. To be specific, the level of CA-125 was 960.08 ± 907.93 U/ml (early) vs. 786.31 ± 1087.37 U/ml (advanced; *p* = .962), CA19-9 was 427.65 ± 519.979 U/ml (early) vs. 291.05 ± 244.72 U/ml (advanced; *p* = .271), D-D was 4.27 ± 5.918 mg/L (early) vs. 1.98 ± 2.177 mg/L (advanced; *p* = .242).

### 3.4. Serum CA-125 and CA19-9 levels in the control group

Same subgroup dividing strategy was performed in the control group. We then explored difference between CA-125, CA19-9 and D-D levels in patients with different stages of unruptured OE, and difference between unilateral and bilateral subgroups. Both levels of CA-125 and CA19-9 were much higher in the bilateral group compared to the unilateral group; however, the difference of CA-125 was not statistically significant (89.90 ± 67.56 U/ml vs. 58.91 ± 59.99 U/ml, *p* = .095), as shown in Table 3. However, the level of CA19-9 was significantly higher in the bilateral group versus the unilateral group (47.09 ± 51.72 U/ml vs. 30.69 ± 29.60 U/ml, *p* = .036).

**Table 3.** Level of CA-125 and CA19-9 in the unruptured OE group.

	CA-125 (U/ml)	CA19-9 (U/ml)
	(mean ± SD)	(mean ± SD)
Side of occurrence		
Unilateral	58.91 ± 59.99	30.69 ± 29.60
Bilateral	89.89 ± 67.56	47.09 ± 51.72
***p* Value**	**0.095**	**0.036**
Stage		
Minimal to mild(I-П)	55.59 ± 30.71	27.13 ± 29.16
Moderate to severe (III-IV)	80.00 ± 77.45	43.00 ± 44.64
***p* Value**	**0.004**	**0.093**

The levels of CA-125 and CA19-9 were higher in patients with advanced stage vs. early-stage disease. As shown in Table 3, the difference in CA-125 levels was statistically significant (80.00 ± 77.45 U/ml vs. 55.59 ± 30.71 U/ml, *p* = .004), while there was no statistically significant difference in the CA19-9 levels (42.99 ± 44.64 U/ml vs. 27.13 ± 29.16 U/ml, *p* = .093).

### 3.5. Performance of serum concentration of CA-125, CA19-9, and D-D in identifying spontaneously ruptured OE

**Figure 1.**
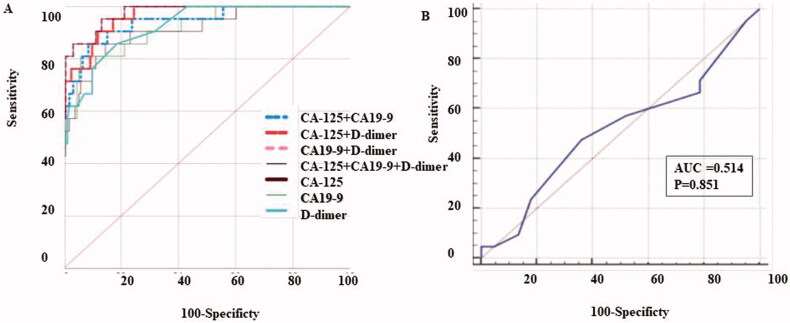
ROC curve for CA-125, CA199, D-dimer and the combined markers for differentiating patients withspontaneously ruptured OE. (A) The AUC value of CA-125, CA199 combined with D-dimer wasthe highest compared with the other groups. (B) The AUC value of OE diameter.

A ROC curve was applied to assess the importance of CA-125, CA19-9, and D-D in differentiating patients with spontaneously ruptured OE. The results were shown in Figure 1(A), Table 4, and Supplemental Table 1.

**Table 4.** Performance of serum concentration of CA-125, CA19-9 and D-dimer in distinguishing the ruptured ovarian endometriosis.

	AUC	AUC (95% CI)	Youden index J	Cut off value	Sensitivity (%)	Specificity (%)	+LR	−LR	*p* Value
CA-125	0.912	0.859 − 0.950	0.7124	108.1	85.71	85.53	5.92	0.17	**<.0001**
CA19-9	0.918	0.867–0.955	0.7503	95.1	80.95	94.08	13.67	0.2	**<.0001**
D-dimer	0.924	0.874–0.959	0.6717	0.6	85.71	81.46	4.62	0.18	**<.0001**
CA-125 + CA19-9	0.945	0.899–0.974	0.7798	–	90.48	87.50	7.24	0.11	**<.0001**
CA-125 + D-dimer	0.964	0.924 − 0.986	0.7789	–	90.48	87.42	7.19	0.11	**<.0001**
CA19-9 + D-dimer	0.975	0.939 − 0.993	0.8240	–	85.71	96.69	25.89	0.15	**<.0001**
**CA-125 + CA19-9 + D-dimer**	**0.976**	**0.940–0.993**	**0.8274**	**-**	**95.24**	**87.50**	**7.62**	**0.05**	**<.0001**
Diameter	0.514	0.436 − 0.591	0.1200	5.7	66.67	21.33	–	–	**.8510**

The AUC showed a statistically significant difference from the null hypothesis (AUC = 0.5), except for OE diameter (shown in Figure 1(B) and Table 4). The AUC for CA-125 was 0.912 (95% CI, 0.859–0.950), with the sensitivity of 85.71% and a specificity of 85.53%. The AUC for CA19-9 was 0.918 (95% CI, 0.867–0.955), with sensitivity and specificity of 80.95% and 94.08%, respectively. Besides, the AUC for D-D was 0.924 (95% CI, 0.874–0.959), with a sensitivity and specificity of 85.71% and 81.46%, respectively. The cut-off values of CA-125, CA19-9, and D-D were calculated as 108.1, 95.1, and 0.6, using MedCal version19.05. Furthermore, the AUC value of the combination of CA19-9 and D-D was 0.975 (95% CI, 0.939–0.993), with the most valuable specificity of 96.69%, LR + of 25.89 and LR − of 0.15, while the sensitivity was 85.71%. The AUC value for the combination of CA-125, CA19-9 and D-D, showed the highest value of 0.976 (95% CI, 0.940–0.993), with the sensitivity of 95.24%, and the highest specificity of 87.50%. The AUC for the diameter of OE was 0.514 (95% CI, 0.436–0.591), with a sensitivity of 66.67%, and a specificity of 21.33%.

We then compared the AUC of each marker alone and in combination using the MedCal software (version19.05). The AUC value for CA-125 was G1, for CA19-9 was G2, and for D-D was called G3. The AUC of the combined groups, CA-125 and CA19-9, CA-125 and D-D, CA19-9 and D-D were named G4, G5, and G6, respectively. For the combined group, CA-125 along with CA19-9 and D-D, the AUC was named as G7. As a result (showed in Supplemental Table 2), there was no significant difference for AUC value between CA125, CA199 and D-D alone. While the difference was significant when G7 was compared with G1 (*p* = .0412), G2 (*p* = .0378) or G3 (*p* = .0461). And also, data showed statistical difference when we compared G6 with G1 (*p* = .0495), G2 (*p* = .0407) or G3 (*p* =.0456). The AUC value obtained by joint detection of 2 or 3 of the serum concentration of CA-125, CA19-9, and D-D was with statistically difference from that of a single marker. However, there was no statistical difference when we compared the AUC value between G7 and G4 (*p* = .0716), G5 (*p* = .2183) or G6 (*p* = .8812) respectively. So conclusion was made that the AUC value for the combined CA-125, CA19-9, and D-D was significantly highest compared with other indicators.

## 4. Discussion

OE is a very common ovarian cyst in women of reproductive age, with patients often showing symptoms as secondary dysmenorrhoea, dyspareunia, and even infertility. While ruptured OE is uncommon, it has not attracted enough attention. Ruptured OE has the ability to cause acute abdominal pain, even severe peritonitis which gradually leads to adhesions and systemic disturbances over the long run and decreases ovarian function [25]. To our best knowledge, few studies have attempted to discriminate ruptured OE from other conditions [23,26]. Yu-Hsin Huang once suggested ruptured OE should be considered in patients who appeared with acute abdominal pain and previously known ovarian cysts with a history of dysmenorrhoea [18]. There was a report claiming the diagnostic rate was as low as 11% for ruptured OE when only the symptoms and physical examinations were considered [19].

Both CA-125 and CA19-9 levels were abnormally elevated in patients with benign tumours[27]. It was widely accepted that CA-125 level was a classic marker for diagnosis of endometriosis because patients with endometriosis had significantly higher levels of CA-125 [14,28,29]. Moreover, the use of serum HE4, CA-125 and CA72-4 were able to distinguish patients with ovarian endometrioma or other benign adnexal masses from those with ovarian malignancy [30]. And elevated levels of CA19-9 in combination with CA-125 had also been examined for evaluation of endometriosis severity [23]. In 2002, Tatsuya Harada *et al.* found that CA19-9 levels were significantly higher in patients with endometriosis compared to patients without endometriosis and were correlated with the severity of endometriosis [15]. Zehra Kurdoglu *et al.* claimed that CA19-9 was a valuable marker for diagnosing endometriosis and was expected to be used to identify patients with severe endometriosis when combining with CA-125 [16]. On the other hand, some studies claimed that patients with endometriosis had significantly higher levels of CA-125; however, CA19-9 alone—or even in combination with CA-125—did not have satisfied results in discriminating patients with or without endometriosis [31,32].

In this study, we focussed on the phenomenon that patients with spontaneously ruptured OE showed unusually high expression of both CA19-9 and CA-125. We found that CA19-9 and CA-125 levels were significantly elevated in the ruptured OE group compared with the unruptured (control) OE group. However, in cases of ruptured endometriosis, no difference was detected in the level of either CA-125 or CA19-9 between subgroups of patients with early or advanced stages. The combination of CA-125 and CA19-9 was proven effective in distinguishing spontaneously ruptured OE from the unruptured one, the AUC for CA19-9 combined with CA-125 was 0.945 (95% CI, 0.899–0.974), with sensitivity and specificity of 90.45% and 87.5%, respectively. Our results were in accordance with the findings which stated that the AUC value for the combined biomarkers, CA-125 and CA19-9, was 0.992 (95% CI, 0.981–1.000) [23].

At the same time, we focussed on elevated D-D levels in patients with ruptured OE. D-D is a secondary product of polymerised fibrin that is degraded by plasmin after blood coagulation. High D-D levels usually indicate fibrinolytic activity. Early in 2003 [20], Hiroshi Fujiwara reported a case with ruptured OE with rapid elevation in serum D-D. When applied immunohistochemical staining, D-D was found deposited in the endometriotic cyst wall. They assumed the rapid elevation of D-D was a result of peritoneal absorption of cyst fluid from ruptured OE which contained fibrin-degraded products, including D-D. In 2015, Kei Tanaka *et al.* [26] focussed on the association between the elevated expression of D-D and ruptured OE. They examined 22 cases of OE. Of these, six cases were ruptured, and 16 cases underwent planned surgery. They clarified that elevated D-D levels were associated with rupture of endometriotic cyst, which was in accordance with our results. However, there was no statistically significant difference in levels of CA-125 between ruptured and unruptured cysts (163.6 ± 126.4 U/ml vs. 86.1 ± 87.5 U/ml, *p* = .12). The authors did not measure the level of CA19-9. We assumed the contradictory result in regard to the level of CA-125 might be caused by limited number of cases in their study.

In this study, the D-D levels in the experimental group were remarkably increased compared to that of the control group. The AUC for the D-D was 0.924 (95% CI, 0.874–0.959), with sensitivity and specificity of 85.71% and 81.46%, respectively. To our knowledge, this is the first assertion that the combination of CA19-9 and D-D was a useful biomarker for the diagnosis of spontaneously ruptured OE. Our results showed that the AUC value for the combined CA19-9 and D-D was 0.975 (95% CI, 0.939–0.993), with the highest specificity of 96.69%, LR + of 25.89, and LR − of 0.15, while the sensitivity was 85.71%. And the combination of CA-125 along with CA19-9 and D-D showed the highest AUC value of 0.976 (95% CI, 0.940–0.993), with the sensitivity of 95.24%, and the highest specificity of 87.50%. From the above result, we suggested choosing the combination of CA19-9 and D-D as a more effective and economical indicator in the clinic. However, from the perspective of differential diagnosis, we suggested CA-125, CA19-9 and D -D should be chosen for the serum test.

This research, however, is subjected to several limitations. First, spontaneously ruptured OE is rare and only 21 patients enrolled in our study, which may cause false-negative result. For example, our result showed no statistically significant difference with respect to OE diameter between patients with spontaneously ruptured OE and the control group (7.50 ± 3.33 U/ml vs. 7.07 ± 2.09 U/ml, *p* = .16), which was discrepant from results of prior literature [23]. Second, our data presented large SDs. The large SD represented the large variation, appropriate and reasonable number of samples can reduce it. However, this study was a retrospective randomised controlled study, in which only 21 cases of ruptured OE patients treated surgically were collected. The low incidence of ruptured OE limited the sample size, ledding to large data dispersion. In addition, based on the clinical data, the serum values of CA-125, CA19-9 of each patient with ruptured OE were objectively discrete, which was also the reason for the large SDs. Similar to our results, Kurdoglu Z *et al* [16] and Dai X *et al* [23] also showed large SDs. However, the difference between the two groups was statistically significant. More cases will be collected at multi-centre institution for more precise results.

## 5. Conclusions

In this study, we found the combination of extremely high levels of CA19-9 along with elevated D-D was expected to be applied as an effective and economical indicator to identify patients with spontaneously ruptured OE pre-operatively. However, from the perspective of differential diagnosis, the combination of CA-125, CA19-9 and D-D is the best choice.

## Supplementary Material

Supplemental Material

## Data Availability

Data generated or analysed during this study are included in this published article. The datasets generated during and/or analysed during this study are available from the corresponding author on reasonable request.
