# Supplementary material for: Extremely high serum CA19-9 level along with elevated D-dimer in assisting detection of ruptured ovarian endometriosis
Source: Ann Med. 2022 Jun 22;54(1):1444–51. doi: 10.1080/07853890.2022.2074534 (PMC9246179; doi:10.1080/07853890.2022.2074534)
Supplement: Supplemental Material [file IANN_A_2074534_SM0398.doc]

**Table S1 General information, pre-operative symptoms and operation findings of ruptured OE**

| Case | Age  (ys) | Known ovarian tumor | | Previous surgery for OE | Latency from abdominal pain to surgery | Abdorminal tendermess | Culdocentesis | Fluid in cul-de-sac  by ultrasound | Surgical procedures |
| --- | --- | --- | --- | --- | --- | --- | --- | --- | --- |
|  |  | time | Size (cm) |  |  |  |  |  |  |
| 1 | 25 | 5 ms | 9 | N | 5 ms | N | NA | N | EE |
| 2 | 30 | 4 ys | 6 | N | >48 hs | Y | NA | N | EE |
| 3 | 32 | 17 ds | 5 | N | >72 hs | Y | NA | N | EE |
| 4 | 26 | 6 ms | 4 | N | 8 hs | Y | NA | N | EE |
| 5 | 28 | Not known | | N | >48 hs | Y | NA | N | EE |
| 6 | 30 | 15 ds | 9 | N | <24 hs | Y | NA | Y | EE |
| 7 | 45 | Not known | | N | 36-48 hs | Y | NA | N | EE |
| 8 | 28 | Not known | | N | 15 hs | Y | EF | Y | EE |
| 9 | 42 | Not known | | N | >72 hs | Y | NA | N | OO |
| 10 | 27 | Not known | | N | 36-48 hs | Y | NA | N | EE |
| 11 | 21 | 1 d | 8 | N | >72 hs | Y | NA | N | EE |
| 12 | 32 | 20 ds | 6 | N | >72 hs | Y | NA | N | EE |
| 13 | 32 | 26 ds | 7 | N | no symptom | N | NA | N | EE |
| 14 | 25 | 3 ys | 5 | N | >72 hs | Y | NA | N | EE |
| 15 | 40 | 7 ms | 4 | N | 24-36 hs | Y | NA | N | EE |
| 16 | 45 | 2 ys | 10 | EE | 36-48 hs | Y | EF | Y | OO |
| 17 | 37 | 1 ms | 8 | N | <24 hs | Y | EF | Y | EE |
| 18 | 37 | 4 ms | 6 | N | >72 hs | Y | NA | N | OO |
| 19 | 29 | 3 ms | 4 | N | <24 hs | Y | NA | N | EE |
| 20 | 27 | 1m | R(6)  L(3) | EE | >72 hs | Y | NA | N | EE |
| 21 | 28 | 6 ms | 8 | N | 36-48 hs | Y | endometriotic fluid | Y | EE |

ys:years; ms:months; ds:days; hs:hours; N:no; Y:yes; NA: not applied; EF: endometriotic fluid ;EE: endometrioma enucleation; OO: oophorectomy.

**Table S2. Comparison of ROC curve for each marker**

| Comparison of ROC curve | P | Comparison of ROC curve | P |
| --- | --- | --- | --- |
| G1 ~ G2 | 0.8716 | G2 ~ G5 | 0.0799 |
| G1 ~ G3 | 0.7765 | G2 ~ G6 | 0.0407* |
| G1 ~ G4 | 0.2928 | G3 ~ G4 | 0.6058 |
| G1 ~ G5 | 0.0799 | G3 ~ G5 | 0.0869 |
| G1 ~ G6 | 0.0495* | G3 ~ G6 | 0.0456* |
| G2 ~ G3 | 0.8892 | G4 ~ G5 | 0.4593 |
| G2 ~ G4 | 0.1516 | G4 ~ G6 | 0.4593 |
| Comparison of ROC curve | P | Comparison of ROC curve | P |
| G1~G7 | 0.0412* | G2~G7 | 0.0378* |
| G3~G7 | 0.0461* | G4~G7 | 0.0716 |
| G5~G7 | 0.2183 | G6~G7 | 0.8812 |

* Represent the difference of statistical significance.

AUC value for CA-125 named as G1, CA19-9 as G2, D-dimer as G3, respectively. AUC curve of the combined CA-125 and CA19-9, CA-125 and D-dimer, CA19-9 and D-dimer were named as G4, G5, G6, respectively.CA-125 along with CA19-9 and D-dimer as was named as G7.
